# Ocular Delivery of Bimatoprost-Loaded Solid Lipid Nanoparticles for Effective Management of Glaucoma

**DOI:** 10.3390/ph16071001

**Published:** 2023-07-13

**Authors:** Sandeep Divate Satyanarayana, Amr Selim Abu Lila, Afrasim Moin, Ehssan H. Moglad, El-Sayed Khafagy, Hadil Faris Alotaibi, Ahmad J. Obaidullah, Rompicherla Narayana Charyulu

**Affiliations:** 1 Department of Pharmaceutics, NGSM Institute of Pharmaceutical Sciences, Nitte (Deemed to be University), Mangalore 575018, India; sandypharama@nitte.edu.in; 2 Department of Pharmaceutics and Industrial Pharmacy, Faculty of Pharmacy, Zagazig University, Zagazig 44519, Egypt; a.abulila@uoh.edu.sa; 3 Department of Pharmaceutics, College of Pharmacy, University of Hail, Hail 81442, Saudi Arabia; a.moinuddin@uoh.edu.sa; 4 Department of Pharmaceutics, College of Pharmacy, Prince Sattam Bin Abdulaziz University, Al-kharj 11942, Saudi Arabia; e.moglad@psau.edu.sa (E.H.M.); e.khafagy@psau.edu.sa (E.-S.K.); 5 Department of Microbiology and Parasitology, Medicinal and Aromatic Plants Research Institute, National Center for Research, Khartoum 2404, Sudan; 6 Department of Pharmaceutics and Industrial Pharmacy, Faculty of Pharmacy, Suez Canal University, Ismailia 41522, Egypt; 7 Department of Pharmaceutical Sciences, College of Pharmacy, Princess Nourah Bint Abdul Rahman University, Riyadh 11671, Saudi Arabia; hfalotaibi@pnu.edu.sa; 8 Department of Pharmaceutical Chemistry, College of Pharmacy, King Saud University, Riyadh 11451, Saudi Arabia; aobaidullah@ksu.edu.sa

**Keywords:** bimatoprost, central composite design, glaucoma, HET-CAM test, solid lipid nanoparticles (SLNs)

## Abstract

Glaucoma is a progressive optic neuropathy characterized by a rise in the intraocular pressure (IOP) leading to optic nerve damage. Bimatoprost is a prostaglandin analogue used to reduce the elevated IOP in patients with glaucoma. The currently available dosage forms for Bimatoprost suffer from relatively low ocular bioavailability. The objective of this study was to fabricate and optimize solid lipid nanoparticles (SLNs) containing Bimatoprost for ocular administration for the management of glaucoma. Bimatoprost-loaded SLNs were fabricated by solvent evaporation/ultrasonication technique. Glyceryl Monostearate (GMS) was adopted as solid lipid and poloxamer 407 as surfactant. Optimization of SLNs was conducted by central composite design. The optimized formulation was assessed for average particle size, entrapment efficiency (%), zeta potential, surface morphology, drug release study, sterility test, isotonicity test, Hen’s egg test-chorioallantoic membrane (HET-CAM) test and histopathology studies. The optimized Bimatoprost-loaded SLNs formulation had an average size of 183.3 ± 13.3 nm, zeta potential of −9.96 ± 1.2 mV, and encapsulation efficiency percentage of 71.8 ± 1.1%. Transmission electron microscopy (TEM) study revealed the nearly smooth surface of formulated particles with a nano-scale size range. In addition, SLNs significantly sustained Bimatoprost release for up to 12 h, compared to free drug (*p* < 005). Most importantly, HET-CAM test nullified the irritancy of the formulation was verified its tolerability upon ocular use, as manifested by a significant reduction in mean irritation score, compared to positive control (1% sodium dodecyl sulfate; *p* < 0.001). Histopathology study inferred the absence of any signs of cornea tissue damage upon treatment with Bimatoprost optimized formulation. Collectively, it was concluded that SLNs might represent a viable vehicle for enhancing the corneal permeation and ocular bioavailability of Bimatoprost for the management of glaucoma.

## 1. Introduction

Glaucoma is a progressive ocular neuropathy that is distinguished by an increment in the intraocular pressure (IOP) predisposing damage to the optic nerve [1]. Glaucoma has been called the silent killer of sight as it begins with minor symptoms and if it left untreated, permanent vision loss can occur. Worldwide, glaucoma is the second leading cause of blindness [2]. Currently, various drugs are available for the treatment of glaucoma. 

Prostaglandins are considered the first-line treatment option for glaucoma. Bimatoprost is a prostaglandin analogue adopted to reduce the elevated intra ocular pressure (IOP) in patients suffering from glaucoma [3]. It is also used for reducing the ocular hypertension [4]. It was officially approved by FDA in the year 2001 for ocular hypertension [5] and later in 2008 for hypotricosis [6]. Bimatoprost has been reported to reduce the increased IOP in patients by boosting the aqueous humor outflow through trabecular meshwork and the uveoscleral pathway [7]. It also increases the production of aqueous humor in the eye that prevents the damage to optic nerve [8]. Bimatoprost has good corneal absorption; it reaches the plasma peak concentration after 10 min and then gets reduced after 1.5 h. Nevertheless, conventional Bimatoprost dosage forms, such as eye drops, show limitations such as lower bioavailability and shorter precorneal residence time. In addition, the commercial formulation of Bimatoprost (Lumigan^TM^) contains benzalkonium chloride, which exerts adverse effects such as eye edema, discomfort, burning, or itching [9]. Furthermore, despite the efficacy of recently adopted approaches, such as nanosponge [10], ocular inserts [11], and intracameral implants [12], in enhancing Bimatoprost corneal accessibility, these treatment strategies usually need a surgical procedure, which might be problematic for patients. 

Ocular drug delivery is considered one of the most difficult tasks confronting pharmaceutical scientists. The poor intraocular bioavailability of conventional ocular delivery systems, caused by high rates of drug dilution and elimination, along with, limited corneal permeability [13,14], usually demand either increased frequency of administration or higher drug levels in the formulation, which may result in problems with patient compliance or potential overdosing. To overcome these problems, novel delivery systems have been introduced, including ocular implants [12], liposomes [15], nanoparticles [16], or in situ gels [17]. Among them, solid lipid nanoparticles (SLNs) have evolved as a viable ocular drug delivery vehicle because of their potential to enhance corneal drug permeation [18]. 

Solid lipid nanoparticle (SLN) are minute colloidal carrier systems with particle size range of 10–1000 nm. Solid lipid nanoparticles represent a viable option for ocular delivery for its inherent properties such as biocompatibility, non-toxicity, and higher stability [19]. In addition, the nanosize range of SLN bestows them with prolonged pre-corneal residence time, controlled drug delivery, enhanced corneal absorption, and thereby, enhanced drug bioavailability. Several studies have addressed the ability of drug-loaded SLNs to effectively cross the corneal epithelium due to its lipophilic properties [19,20,21]. Furthermore, SLN offers the advantages of the ability to encapsulate both hydrophilic and lipophilic drugs, high drug loading efficiency, and ease of large-scale production [22,23].

In this study, Bimatoprost-loaded SLNs were formulated to improve the therapeutic effectiveness of drug for the management of glaucoma. Central composite design (CCD) was adopted for the optimization of Bimatoprost-loaded SLNs investigating the impact of two independent factor, drug: lipid ratio (*w*:*w*) and sonication time (min) on two product responses; particle size and entrapment efficiency. Further, the optimized Bimatoprost-loaded SLNs was subjected for evaluation parameters such as mean particle size, zeta potential, TEM analysis, drug release study, sterility test, isotonicity test, HET-CAM test and histopathology studies.

## 2. Results and Discussion

### 2.1. Preformulation Studies

**Figure 1:**
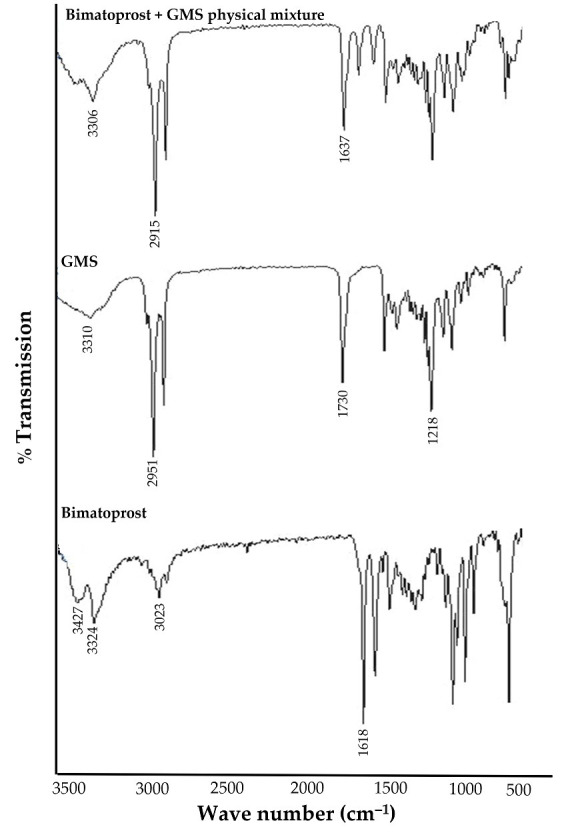
FTIR spectra of Bimatoprost, GMS, and their physical mixture.

FTIR studies were carried out to assess the compatibility of drug with lipid used for preparing the Bimatoprost-loaded SLNs. The FTIR spectra of pure Bimatoprost, glyceryl monostearate (GMS) and physical mixture of drug and GMS are depicted in Figure 1. The FTIR spectrum of Bimatoprost showed characteristic peaks at 3324, 1618, 3427 and 3023 cm^−1^ corresponding to O-H stretching, N-C=O (amide) stretching, N-H (secondary amine) stretching and aliphatic C-H (stretching) groups. For GMS, the FTIR spectrum showed absorption peaks at 1218, 1730, 2951 and 3310 cm^−1^ demonstrating the characteristic function groups of C-O-C (ether) stretching, C=O (carbonyl), aliphatic C-H stretching and O-H (hydroxyl) groups, respectively. Of interest, the FTIR spectrum obtained for physical mixture of Bimatoprost and GMS exhibited prominent peaks at 3306, 1637, and 2915 cm^−1^ corresponding to the O-H (alcohol) stretching, N-C=O (amide) stretching, aliphatic C-H stretching functional groups. From the FTIR interpretation, it was evident that there was no interaction between the drug and lipid. Therefore, this study claimed that Bimatoprost was compatible with GMS. 

### 2.2. Preparation of Bimatoprost-Loaded SLNs 

A central composite statistical approach was adopted for the formulation of Bimatoprost-loaded SLNs and to study the impact of two independent factors; namely drug:lipid ratio and sonication time, on product characteristics. The influence of these factors on particle size and entrapment efficiency (%) was investigated as depicted by the 3D surface plots. Considering these two factors, a total of 13 formulation trials at different levels of independent factors were prepared. All the fabricated formulation trials were then evaluated for particle size and entrapment efficiency (%). The optimization was aimed at minimizing the particle size and maximizing the entrapment efficiency (%). The central composite design generated trails with variable particle sizes and entrapment efficiency (%) illustrated in the Table 1.

#### 2.2.1. Effect of Formulation Variables on Particle Size

Generally, nanoparticles having an average particle size smaller than 200 nm are thought to be optimum for ocular delivery because of their enhanced penetration through the corneal barriers [24]. In this study, the particle size of all Bimatoprost-loaded SLNs was ranging from 178.6 ± 9.3 to 322.3 ± 17.6 nm. The data for particle size (R_1_) were fitted into several polynomial models. ANOVA analysis (Table S1) revealed that R_1_ was best fitted in a quadratic response surface model (Equation (1)).

Particle size (R_1_) = +190.00 + 12.39 A − 25.24 B − 13.12 AB + 55.99 A^2^ + 13.91 B^2^ (1)

**Figure 2:**
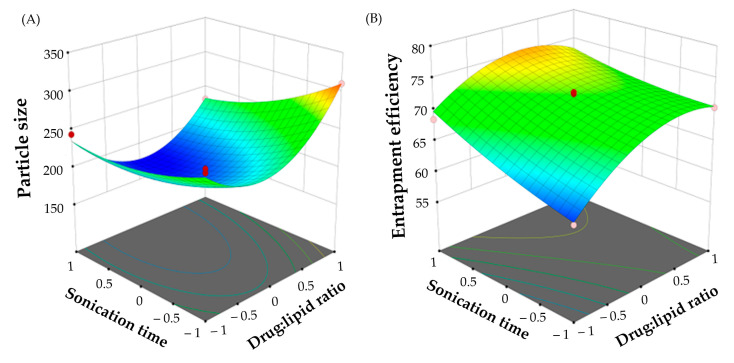
3D surface plot of (**A**) Particle size, and (**B**) entrapment efficiency percentage.

The impact of independent variables on particle size was analyzed by the 3D surface plot obtained from the data of study with the Design Expert software (Figure 2A). The surface plots obtained with particle size showed a synergistic effect of drug:lipid ratio on SLNs particle size. Increasing lipid ratio from 1:1 to 1:5 resulted in a significant increase of particle size. By fixing the sonication time, the particle size of SLNs prepared with a drug:lipid ratio of 1:5 (F10; 310.6 ± 19.5 nm) was remarkably higher than that prepared at 1:1 drug:lipid ratio (F2; 269.8 ± 18.2 nm). This effect might be ascribed to the increase in the viscosity of the organic phase at higher polymer concentrations, which in turn, would increase the tendency of the lipid to coalesce, leading to the formation of larger nanoparticles. Similar results were obtained by Tiwari et al. [25] who reported the positive effect of increasing lipid content on the particle size of terbinafine hydrochloride-loaded SLNs.

Sonication energy played a crucial role in formation of the emulsion, and thus significantly affected the particle size of SLNs. The sonication energy was varied by altering the sonication period (5 to 15 min) while maintaining a constant power. Herein, sonication time was found to exert an antagonistic effect on particle size of SLNs. At fixed drug:lipid ratio, the particle size of Bimatoprost-loaded SLNs dramatically decreased from 310.6 ± 19.5 nm (F10) to 232.6 ± 11.6 nm (F11) as the sonication time increased from 5 min to 15 min. Collectively, these results revealed the pronounced effects of formulation variables on particle size.

#### 2.2.2. Effect of Formulation Variables on Entrapment Efficiency Percentage

Entrapment efficiency is a crucial measure for characterizing solid lipid nanoparticles. In order to obtain an optimum encapsulation efficiency, several factors, including drug:lipid ratio and sonication time, were varied, and the entrapment efficiency was calculated. As depicted in Table 2, the percentage entrapment efficiency fluctuated from 60.8 ± 1.1 to 78.6 ± 1.3%. The data for entrapment efficiency (R_2_) were fitted into several polynomial models. ANOVA analysis (Table S1) revealed that R_2_ was best fitted in a quadratic response surface model (Equation (2)).

Entrapment efficiency (%) (R_2_) = +71.98 + 3.10 A − 2.70 B − 1.30 AB − 3.83 A^2^ + 0.72 B^2^ (2)

In addition, the influence of independent variables on entrapment efficiency was represented by the 3D surface plot (Figure 2B). It was evident that drug:lipid ratio exerted a positive effect on drug entrapment within SLNs. The entrapment efficiency rose from 61.4 ± 1.9% (F2) to 72.3 ± 1.7% (F10) when the drug-to-lipid weight ratio was changed from 1:1 to 1:5, indicating that the greater the lipid ratio, the higher the entrapment efficiency. This pattern was consistent with previous literature [26,27]. This synergistic effect of lipid content on drug entrapment efficiency might be explained, on the one hand, to the availability of higher space to accommodate more drug at higher lipid content [28], and on the other hand, to the elevated viscosity of the medium at higher lipid content, which result in rapid solidification of the nanoparticles, and thereby, constrain the leakage of drug to the external medium [29]. 

Similarly, sonication time has been verified to exert a synergistic effect on drug entrapment within SLNs. At fixed drug:lipid ratio, the entrapment efficiency percentage was increased as the sonication time increased from 5 min to 15 min. A higher entrapment efficiency (68.5 ± 1.1%; F3) was achieved when Bimatoprost-loaded SNLs dispersion was processed by probe-sonication for 20 min, compared to those sonicated for 5 min (F2; 61.4 ± 1.9%). This effect might be ascribed to the increased drug solubility in the lipid core upon increasing sonication time, which in turn, would increase the entrapment efficiency [21]. Similar findings were reported by Nair et al. who emphasized the positive effect of increasing sonication time on the entrapment of clarithromycin within solid lipid nanoparticles [30].

#### 2.2.3. Numerical Optimization of Bimatoprost-Loaded SLNs

In order to obtain an optimized Bimatoprost-loaded SLN formulation, a numerical optimization analysis was carried out using Design-Expert^®^ software employing the desirability function. The suggested formulation variables for the fabrication of the optimized Bimatoprost-loaded SLN formulation, obtained at a desirability of 0.936, was drug:lipid ratio of 1:3, and a sonication time of 20 min. The prepared optimized Bimatoprost-loaded SLNs was found to fulfill the requisites of a minimum particle size and maximum entrapment efficiency set by the design constrains for an optimum formulation. The estimated vesicle size was 183.3 ± 13.3 nm, and the % EE was 71.8 ± 1.1%, which were comparable to the predicted values for vesicle size and % EE (178.7 nm, and 75.4%, respectively).

### 2.3. Evaluation of Optimized Formulation of Bimatoprost SLNs

#### 2.3.1. Particle Size and PDI

**Figure 3:**
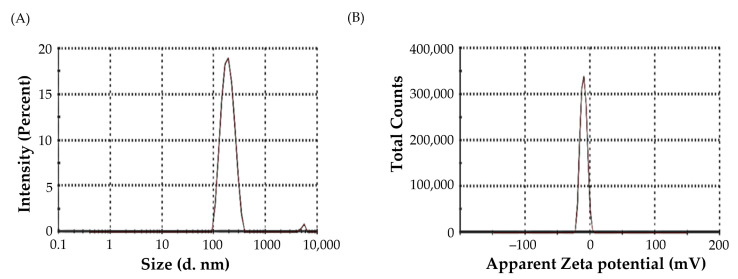
(**A**) Particle size, and (**B**) Zeta potential of optimized Bimatoprost-loaded SLNs.

The average particle size of optimized Bimatoprost-loaded SLNs was found to be 183.3 ± 13.3 nm (Figure 3A), which is considered within the optimum size range (<200 nm) for ocular administration [24]. Polydispersity index (PDI) is another important parameter for characterizing nanodispersions. PDI is considered a measure of the heterogeneity of a sample based on size. Generally, colloidal particles with PDIs values less than 0.3 denotes homogenous size distribution. In this study, the PDI of optimized Bimatoprost-loaded SLNs was 0.205, indicating high particle homogeneity [31].

#### 2.3.2. Zeta Potential

The magnitude of the zeta potential of colloidal particles provides an indication of their physical stability. Colloidal particles with either large positive or negative zeta potential tend to repel each other, and thereby, enhance the overall stability of the colloidal system [32]. The zeta potential of optimized Bimatoprost-loaded SLNs was found to be −9.96 ± 1.2 mV (Figure 3B). This relatively negatively charged surface of the prepared SLNs, along with the lower PDI value, is assumed to participate to the physical stability of formulated SLNs.

#### 2.3.3. TEM Study

**Figure 4:**
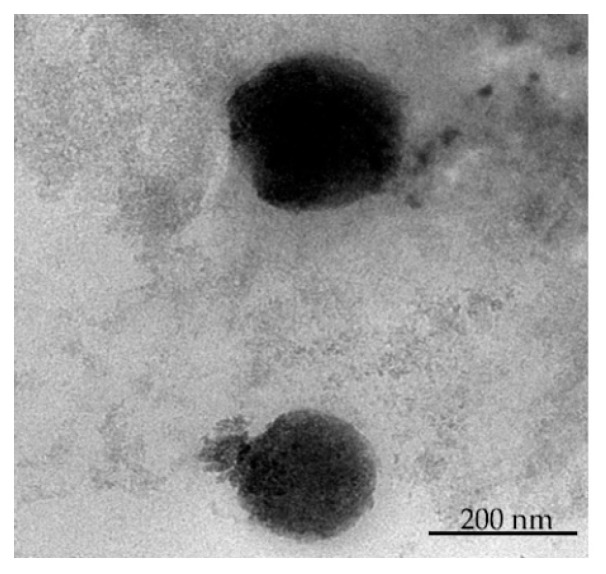
TEM analysis of optimized Bimatoprost-loaded SLNs.

The surface morphology and particle size distribution of optimized formulation were evaluated by TEM analysis. As depicted in Figure 4, the optimized drug-loaded SLNs exerted a discrete structure with a nearly smooth surface. In addition, particle size of the optimized SLNs determined by TEM was in the range of 150–200 nm, which is close to that determined by dynamic light scattering technique.

### 2.4. In Vitro Drug Release Study

**Figure 5:**
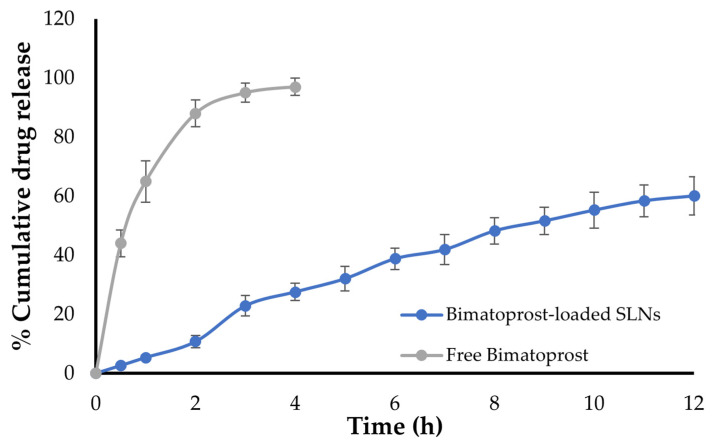
Drug release profile of optimized Bimatoprost SLNs.

The in vitro dissolution/release profiles of both free Bimatoprost and optimized drug-loaded SLNs were illustrated in Figure 5. The study was conducted using simulated tear fluid (STF) as dissolution media using Franz diffusion cell. As shown in Figure 5, free Bimatoprost was released immediately from Bimatoprost solution with more than 95% of the drug released within the first 4 h. On the other hand, entrapment of Bimatoprost within SLNs remarkably prolonged the drug release for up to 12 h. Of interest, Bimatoprost-loaded SLNs showed a biphasic release pattern, with more than 25% of entrapped Bimatoprost was released from the SLNs during the first 3 h, followed by a sustained release over 12 h, with up to 60% of drug released at the end of release study. The initial rapid drug release from SLNs might be accounted for the ready dissolution of drug molecules adsorbed at the surface of SLNs, while the subsequent sustained drug release might be ascribed to the increase in the diffusion pathlength for drug molecules that are efficiently entrapped within the inner core of SLNs.

The in vitro release profile of the optimized Bimatoprost-loaded SLNs was fitted to various kinetics models, i.e., zero order, first order, Highuchi release and Kores-peppas models. Fitting the release data into these release kinetic models demonstrated that Bimatoprost release from SLNs followed the Higuchi kinetics, indicating a diffusion-controlled mechanism (Table S2).

### 2.5. Stability Studies

The stability of colloidal dispersions is considered a crucial parameter for ensuring their satisfactory use. The stability data for optimized Bimatoprost-loaded SLNs are summarized in Table S3. The optimized formula was found to be stable upon storage as manifested by slight changes in the particle size, zeta potential and percentage drug entrapment at 8 weeks post storage compared to fresh samples.

### 2.6. Sterility Test

Generally, all ophthalmic preparations must fulfill a variety of safety and effectiveness standards, including product sterility. Sterility is defined as the lack of live microbial contamination, which if present in such formulations, might result in eye infections with potentially serious consequences. As summarized in Table 2, sterility testing confirmed the absence of any signs of either bacterial or fungal growth with the optimized formulation when compared with positive control. These results that optimized Bimatoprost SLNs formulation meets the criterion of sterility required for ophthalmic preparations.

### 2.7. Isotonicity Test

**Figure 6:**
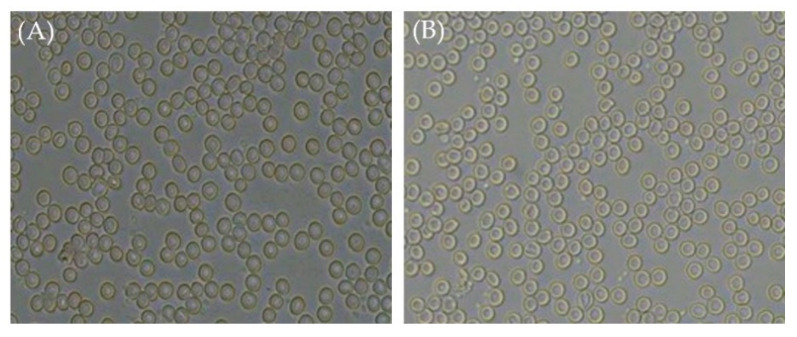
Microscopical images of RBCs treated with (**A**) marketed eye drops and (**B**) optimized Bimatoprost-loaded SLNs. Magnification 400x.

Isotonicity is another important feature of ophthalmic products. Isotonicity must be maintained to avoid tissue damage or eye discomfort [33]. Isotonic solutions keep blood cells intact, whereas hypotonic solutions cause cell bulging and hypertonic solutions cause cell shrinkage. As shown in Figure 6, microscopical examination of red blood cells (RBCs) upon treatment with few drops of optimized Bimatoprost-loaded SLNs revealed the absence of any alteration in RBCs shape, and the effect of drug-loaded SLNs on RBCs was comparable to that of marketed eye drops on RBCs. These results confirmed the safety of drug-loaded SLNs for ophthalmic application.

### 2.8. Ocular Irritation (HET-CAM) Test

**Figure 7:**
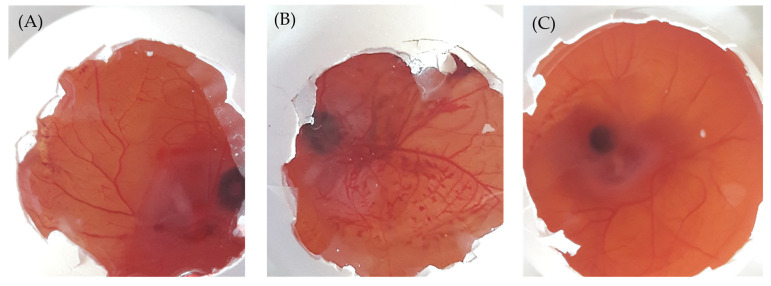
HET-CAM images after application of (**A**) 0.9% saline solution, (**B**) 1% sodium dodecyl sulfate, and (**C**) Optimized formulation. Magnification 10x.

In this study, the Hen’s Egg Test on the ChorioAllantoic Membrane (HET-CAM) has been adopted as an alternative method to animal (Draize eye test) experimentation to evaluate the possible ocular irritation elicited by Bimatoprost-loaded SLNs [34]. In HET-CAM test, the optimized SLNs formulation was tested on chorioallantonic membrane, which mimics the structure of ocular membrane. The potential ocular irritancy of test formulation was investigated visually via recording the morphological changes in the CAM such as vascular lysis, coagulation and hemorrhage [35]. The mean irritation scores of test formulation, positive and negative controls were represented in Table 3. As illustrated in Table 3, the negative control (0.9% NaCl solution) had mean irritation score of ~ 0 with no signs of any ocular irritation (Figure 7A). On the other hand, the mean irritation score of positive control (1% SDS) was found to be 15.68, reflecting the induction of severe irritation as manifested by blood vessel lysis and severe hemorrhage in the CAM (Figure 7B). Of interest, the optimized formulation showed a mean irritation score of 0.02 with no signs of any ocular irritation in the CAM when compared with positive control (Figure 7C). Therefore, the study claimed that Bimatoprost-loaded SLNs were safe for ocular administration since it induced no signs of ocular irritation. 

### 2.9. Ex Vivo Histopathology Study

**Figure 8:**
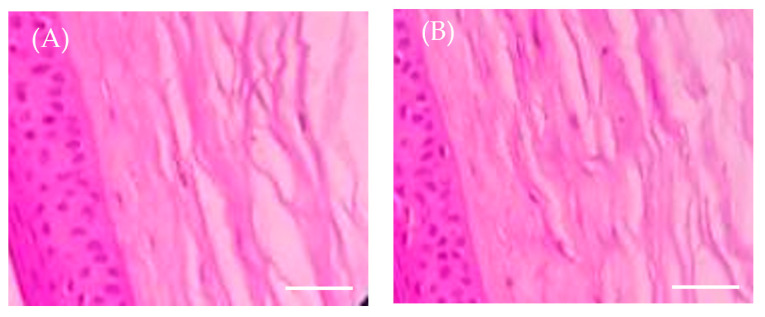
Ex vivo histopathology images of corneal membrane treated with (**A**) 0.9 NaCl solution and (**B**) optimized Bimatoprost-loaded SLNs. Scale bare 25 μm.

Histopathology studies were performed to address the occurrence of any changes or damage to corneal tissue upon optimized formula application. The histopathology images were depicted in Figure 8. The cornea treated with 0.9% sodium chloride solution (negative control) exhibited no signs of tissue injury (Figure 8A). Interestingly, cornea treated with optimized Bimatoprost-loaded SLNs showed no detrimental impact on corneal epithelium, endothelium, or stroma, indicating no or mild corneal toxicity. The corneal epithelium was intact and was found to be attached to Bowman’s membrane (Figure 8B). These findings confirmed that the optimized formulation was non-toxic to the corneal membrane.

## 3. Materials and Methods

### 3.1. Materials

Bimatoprost was obtained from Dr. Pradeep Reddy Laboratories (Hyderabad, India). Glyceryl monostearate (GMS), chloroform, dimethyl sulfoxide (DMSO), sodium chloride, dibasic sodium phosphate and benzalkonium chloride were procured from Loba Chemie (Mumbai, India).

### 3.2. Drug-Lipid Compatibility by FTIR (Fourier Transform Infrared) Spectroscopy 

For preparing Bimatoprost-loaded SLNs, glyceryl monostearate (GMS) was selected as lipid carrier and its compatibility with the drug was assessed by FTIR spectroscopy using IR spectrophotometer (Bruker, Billerica, MA, USA) [36]. The study involved getting the IR peaks of drug, lipid and their physical mixture and the obtained peaks were referred with standards and interpretation was done to confirm whether the drug and lipid used in the study are compatible with one another or not.

### 3.3. Preparation of Bimatoprost-Loaded SLNs 

Bimatoprost-loaded SLNs were fabricated by solvent evaporation method and ultrasonication technique using probe sonicator [37]. Briefly, accurately weighed amount of the drug and GMS were dissolved in chloroform/dimethyl sulfoxide mixture (1:1 *v*/*v*). In another beaker, 5% of poloxamer 407 was dissolved in 15 mL of distilled water by magnetic stirring. To this surfactant solution, the organic solvents containing drug and GMS mixture was slowly added drop by drop and the mixture was heated at 60–70 °C to evaporate the organic solvents followed by magnetic stirring. The SLNs dispersion was stirred for time period of 5 h. Finally, SLNs dispersion was subjected to probe sonication for further particle size reduction. The resultant dispersion was sterilized by filtering through a 0.22 μm pore size membrane and kept under refrigeration (2–8 °C) until use.

### 3.4. Optimization of Bimatoprost-Loaded SLNs

A central composite design (CCD) was used for optimization of Bimatoprost-loaded SLNs by varying drug:lipid ratio and sonication time as independent factors. The dependent factors considered were particle size (R_1_) and percentage entrapment efficiency (R_2_). The impact of independent formulation variables on dependent responses was investigated from three-dimensional (3D) surface plots constructed from the design study. Optimization study was done with Design Expert^®^ software (Version 12; Stat-Ease, Inc., Minneapolis, MN, USA) which provided 13 experimental runs at different drug:lipid ratios and sonication times (low, medium and high). All the 13 formulations were evaluated for the particle size and entrapment efficiency (%) and these data were analyzed for final optimization. The dependent and independent factors with actual levels of factorial design were represented in Table 4.

### 3.5. Evaluation of Bimatoprost-Loaded SLNs

#### 3.5.1. Particle Size, Polydispersity Index (PDI) and Zeta-Potential

Particle size and PDI of all the SLNs were measured with zeta sizer (Malvern Instruments Ltd., Worcestershire, UK). Few drops of the formulation were placed in a cuvette cell and exposed to the laser beam. The intensity of scattered light were adopted to estimate the particle sizes of the samples [38]. 

The zeta potential was measured for the optimized formulation with zeta sizer (Malvern Instruments Ltd., Worcestershire, UK) [39].

#### 3.5.2. Entrapment Efficiency (%)

Entrapment efficiency (%) gives information about the percentage of drug encapsulated within the lipid nanoparticles. The entrapment efficiency (%) of Bimatoprost-loaded SLNs was estimated indirectly by separating Bimatoprost-loaded SLNs from the supernatant containing free Bimatoprost by centrifugation at 15,000 rpm for about 30 min. The concentration of free drug in supernatant was quantified spectrophotometrically at 294 nm. The entrapment efficiency (%) was calculated using the following formula [40]:

$$\%EE=\frac{Total\;initial\;amount\;of\;drug-Free\;drug}{Total\;initial\;amount\;of\;drug}$$

#### 3.5.3. Transmission Electron Microscopy (TEM) Study

The surface morphology, particle size range and the particle size distribution of optimized formulation was determined by TEM study. For this purpose, few drops of formulation were placed on 300 mesh copper grid (copper coated film) and allowed to air dry for 10 min. For staining the sample, 2% *w*/*v* of phosphotungstic acid was applied for about 2 min, and excess of liquid was dried with filter paper the sample was subsequently placed in transmission electron microscope. Later, TEM photographs were taken and examined for size distribution analysis [41]. 

### 3.6. Stability Study

The stability study of optimized formulation was carried out by storing SLNs at 4 ± 1 °C in glass vials for 2 months. The alteration in particle size, zeta potential and/or entrapment efficiency was assessed at entrapment efficiency at 0, 4, and 8-weeks following storage [42].

### 3.7. In Vitro Drug Release Study

The in vitro drug release from optimized formulation was conducted using modified Franz diffusion cell at 37 ± 0.5 °C under stirring at 50 rpm. Briefly, a dialysis membrane, previously soaked in the simulated tear fluid (STF) for overnight, was fitted in-between the receptor and donor compartments of diffusion cell. The receptor compartment was filled with 25 mL of freshly prepared STF (pH 7.4). A definite weight of formulation (containing 2 mg of drug) was placed in the donor compartment. Drug release study was lasted for 12 h during which aliquot samples (1 mL) from receptor compartment were taken at scheduled time points and were replaced with same amounts of STF to maintain sink condition. The samples were diluted with STF and their absorbances were measured at 294 nm using spectrophotometer. The obtained data was further converted to percentage cumulative drug release (% CDR).

Finally, drug release data of optimized formulation was fitted into different drug release kinetic models such as zero order, first order, Highuchi release and Kores-peppas release studies. The best kinetic model was predicted with that highest regression value (R^2^) obtained [43].

### 3.8. Sterility Test

Sterility is one of the vital characteristic features of ophthalmic formulation. Sterility of optimized formulation was evaluated by following the direct inoculation method. The procedure of the method was described in Indian Pharmacopoeia (IP). The formulation was tested against two species of bacteria (*Staphylococcous aureus* and *Bacilus subtilus)* and two fungi species (*Candida albicans* and *Asperges brasiliensis*) inoculated in fluid thioglycolate medium for bacteria and soyabean casein digest medium for fungi in test tubes. The inoculated test tubes were incubated for a period of 7 days. After 7 days of incubation, the samples were tested for sterility by comparing the test sample with positive and negative controls [44]. 

### 3.9. Isotonicity Test

Isotonicity is one of the important characteristics of ophthalmic formulation. The formulation should be isotonic with blood for safe administration. Isotonicity of the formulation was checked with human blood. Few drops of optimized formulation were mixed with human blood and microscopically examined for the physical appearance of RBCs. The study was compared with the marketed eye drops of Bimatoprost to check if there is any variation in the RBCs shape and also to visualize any shrinkage and bulginess of RBCs [45].

### 3.10. Ocular Irritancy by HET-CAM Test

The ocular irritancy of the optimized formulation was examined by Hen’s egg test-chorioallantoic membrane (HET-CAM). The chorioallantoic membrane (CAM) was developed in freshly collected fertile eggs. The eggs were kept on a tray and incubated at 37 ± 0.5 °C and at relative humidity of 55 ± 5% for a period of 5 days. Each day, the eggs were rotated manually 5 times for complete fertilization. After 5 days, the eggs were examined for the growth of embryo by candling them under flashlight. The non-viable eggs were discarded, while the viable eggs were further incubated till day 10. On the 10th day, all the eggs were removed from the incubator, the eggshell was broken with a blade without harming the embryo (CAM) present inside the egg. CAM of eggs was treated with 0.3 mL of optimized formulation, 0.3 mL of Sodium dodecyl sulfate (1%) as positive control and 0.3 mL of 0.9% Sodium chloride (NaCl) as negative control. CAM of eggs was observed visually for a time period of 5 min for the irritation signs like lysis of blood vessels, coagulation and hemorrhage. The mean irritation scores of all respective samples were determined and based on the irritation score value, the irritancy level was determined [46]. The irritation score was estimated using the following formula [9] and the level of irritancy with standard score range is represented in Table 5.

$$Irritation\;score\left(IS\right)=\frac{301-Hemorrhage}{300}\times 5+\frac{301-lysis}{300}\times 7+\frac{301-coagulation}{300}\times 9$$

where, all the values of coagulation, lysis and hemorrhage are recorded in seconds.

### 3.11. Ex Vivo Histopathology Study

Histopathology study was carried out to evaluate the effect of formulation on the corneal tissue and its integrity. Goat corneas were isolated from the whole goat eyes that are procured from the slaughterhouse. Corneal layer with sclera tissue was carefully removed by using sterile scissors and preserved in normal saline to retain the tissue consistency. The corneas were treated with optimized formulation (Test). For comparison purpose, untreated cornea was used as negative control. All the corneas were immersed in 8% formalin and left for about 8 h. Later, the corneas were hydrated by washing with ethyl alcohol and distilled water. The corneal tissue was stained with hematoxylin and eosin and the images of all the corneas were taken and the assessment was to check any morphological changes found in corneas treated with test sample [47]. 

### 3.12. Statistical Analysis

All values were expressed as mean ± standard deviation. An unpaired Student’s *t*-test and one-way ANOVA (GraphPad Software, Version 6, San Diego, CA, USA) were used for statistical analysis. A *p* value less than 0.05 implies a significant difference.

## 4. Conclusions

In this study, Bimatoprost-loaded SLNs were developed and optimized for ocular administration. The prepared drug loaded SLNs exhibited smooth surfaces with nano-scale size range. In vitro drug release study revealed the sustained release properties of optimized formulation. Sterility test confirmed the sterile nature of formulation with no signs of any microbial growth. Most importantly, HET-CAM test nullified the irritancy potential of optimized formulation against chorioallantonic membrane, claiming the non-irritant nature of the formulation. In addition, histopathology study verified the tolerability of test formulation by corneal tissue as manifested by the absence of any signs of corneal tissue damage. Collectively, it was concluded that Bimatoprost-loaded SLNs might represent a potential and promising ocular drug delivery approach for the effective management of glaucoma.

## Figures and Tables

**Table 1:** Central composite design batches with results of dependent factors for Bimatoprost SLNs.

Formula	A: Drug:Lipid Ratio (*w*:*w*)	B: Sonication Time (min)	R_1_: Particle Size (nm)	R_2_: Entrapment Efficiency (%)
1	0	0	184.7 ± 11.4	72.7 ± 2.4
2	−1	−1	269.8 ± 18.2	61.4 ± 1.9
3	−1	1	244.3 ± 13.7	68.5 ± 1.1
4	0	0	188.6 ± 9.5	71.4 ± 2.1
5	0	0	185.5 ± 7.6	72.8 ± 1.8
6	0	0	192.8 ± 11.1	70.4 ± 1.6
7	0	0	198.4 ± 12.4	72.6 ± 1.9
8	0	−1.41421	248.2 ± 18.6	69.7 ± 0.9
9	0	1.41421	178.6 ± 9.3	78.6 ± 1.3
10	1	−1	310.6 ± 19.5	70.4 ± 2.5
11	1	1	232.6 ± 11.6	72.3 ± 1.7
12	1.41421	0	322.3 ± 17.6	69.3 ± 0.8
13	−1.41421	0	272.8 ± 12.3	60.8 ± 1.1

**Table 2:** Sterility test results for optimized formulation.

Test Microorganism	Positive Control	Negative Control	Optimized Formulation
*Staphylococcous aureus*	+	−	−
*Bacilus subtilus*	+	−	−
*Candida albicans*	+	−	−
*Asperges brasiliensis*	+	−	−

where: + sign indicates microbial growth, while − sign indicates no microbial growth.

**Table 3:** Mean irritation scores of HET-CAM test.

Test Compound	Mean Irritation Score	Inference
Negative control (0.9% NaCl)	0.01 ± 0.01	No irritation
Optimized Bimatoprost SLNs	0.02 ± 0.01	No irritation
Positive control (1% SDS)	15.68 ± 0.78	Severe irritation

**Table 4:** Central composite design with coded values of factors.

Factors	Levels, Actual (Coded)		
Independent Factors	−1 (Low)	0 (Medium)	+1 (High)
A = Drug:lipid ratio (*w*:*w*)	1:1	1:3	1:5
B = Sonication time (in)	5	10	15
Dependent factors			
Particle size (nm) (R_1_)			
Entrapment efficiency % (R_2_)			

**Table 5:** HET-CAM test irritation score chart.

Irritation Score	Mean Score Value	Level of Irritation
0–0.9	0	No irritation
1–4.9	1	Slight irritation
5–8.9	2	Moderate irritation
9–21	3	Severe irritation

## Data Availability

Data is contained within the article and Supplementary Material.

## Supplementary Materials

The following supporting information can be downloaded at: https://www.mdpi.com/article/10.3390/ph16071001/s1, Table S1: Results of statistical analysis of all dependent variables R_1_ and R_2_; Table S2: In vitro release kinetics data of optimized Bimatoprost-loaded SLNs; Table S3: Stability study for optimized Bimatoprost-loaded SLNs.
