# Supplementary material for: Ocular Delivery of Bimatoprost-Loaded Solid Lipid Nanoparticles for Effective Management of Glaucoma
Source: Pharmaceuticals (Basel). 2023 Jul 13;16(7):1001. doi: 10.3390/ph16071001 (PMC10385266; doi:10.3390/ph16071001)
Supplement: Supplementary file 1 [file pharmaceuticals-16-01001-s001.zip › pharmaceuticals-2464050-supplementary.pdf]

Table S1. Results of statistical analysis of all dependent variables

Source	Y_1		Y_2	
	F-Value	*p*-Value	F-Value	*p*-Value
Model	81.94	<0.0001	34.60	< 0.0001
A: Drug:lipid ratio	17.27	0.0043	52.35	0.0002
B: Sonication time	71.69	<0.0001	39.60	0.0004
Lack of Fit	3.82	0.114	1.78	0.2898
R^2 analysis				
R^2	0.9832		0.9611	
Adjusted R^2	0.9712		0.9333	
Predicted R^2	0.9046		0.8158	
Adequate precision	23.9795		21.0007	

Table S2: *In vitro* release kinetics of optimized Bimatoprost-loaded SLNs

Formulation	Zero-order	First-order	Higuchi	Kores-Peppas
	R^2			
Bimatoprost-loaded SLNs	0.9753	0.7468	0.9917	0.9705

Table 3: Stability study of optimized Bimatoprost-loaded SLNs

Parameters	Day 0	Storage for 4 weeks at 4 ± 1 ˚C	Storage for 8 weeks at 4 ± 1 ˚C
Particle size (nm)	183.3 ± 13.3	189.6 ± 17.2	196.5 ± 19.8
Zeta potential (mV)	– 9.9 ± 1.2	– 9.4 ± 1.6	– 9.1 ± 1.9
Entrapment efficiency (%)	71.8 ± 1.1	70.1 ± 0.9	69.7 ± 1.7

Data represent the mean ± SD
